# Progressive, refractory macrophage activation syndrome as the initial presentation of anti-MDA5 antibody positive juvenile dermatomyositis: a case report and literature review

**DOI:** 10.1186/s12969-022-00675-w

**Published:** 2022-02-22

**Authors:** J. Alex Stewart, Theresa Price, Sam Moser, Dolores Mullikin, Angela Bryan

**Affiliations:** 1 Tripler Army Medical Center Department of Pediatrics, Honolulu, HI USA; 2 Madigan Army Medical Center Department of Pediatrics, Honolulu, HI USA

**Keywords:** Hyperferritinemia, Myositis, Atrial fibrillation, Idiopathic inflammatory myopathy, Myocarditis, Rheumatologic disease, Immunosuppressant, Systemic juvenile idiopathic arthritis

## Abstract

**Background:**

Macrophage activation syndrome (MAS) is a severe and under-recognized complication of rheumatologic diseases. We describe a patient who presented with rapidly progressive, refractory MAS found to have anti-MDA5 antibody Juvenile Dermatomyositis (JDM) as her underlying rheumatologic diagnosis.

**Case presentation:**

We describe a 14-year-old female who at the time of admission had a history of daily fevers for 6 weeks and an unintentional sixteen-pound weight loss. Review of systems was significant for cough, shortness of breath, chest pain, headaches, sore throat, muscle aches, rash, nausea, and loss of appetite. An extensive initial workup revealed findings consistent with an autoimmune process. While awaiting results of her workup she had clinical decompensation with multi-organ system involvement including pancytopenias, interstitial lung disease, hepatitis, cardiac involvement, gastrointestinal distension and pain, feeding intolerance, extensive mucocutaneous candidiasis, and neuropsychiatric decline. Due to her decompensation, significant interstitial lung disease, and likely underlying rheumatologic condition she was started on high dose pulse steroids and mycophenolate. An MRI was performed due to her transaminitis and shoulder pain revealing significant myositis. Intravenous immunoglobulin was then initiated. The myositis antibody panel sent early in her workup was significant for anti-MDA5 and anti-SSA-52 antibodies. Despite high dose pulse steroids, mycophenolate, and IVIG, her disease progressed requiring escalating therapies. Ultimately, she responded with resolution of her MAS as well as significant and steady improvement in her feeding intolerance, interstitial lung disease, cardiac dysfunction, myositis, arthritis, and cutaneous findings.

**Conclusions:**

JDM in the pediatric patient is rare, as is MAS. In patients with complex rheumatologic conditions and lack of response to treatment, it is important to continually assess the patient’s clinical status with MAS in mind, as this may change the treatment approach. Without proper recognition of this complication, patients can have a significant delay in diagnosis leading to life-threatening consequences.

## Background

Juvenile Dermatomyositis (JDM) is a subtype of the idiopathic inflammatory myopathies (IIM), a group of diseases characterized by muscle inflammation and resulting weakness. JDM is the most common IIM in children with an estimated incidence in the United States of 2.5–4.1 per one million children between 1995 and 1998 [1]. The peak age of onset is approximately 7 years old and is more common in girls than boys. Like many rheumatologic diseases, it is not uncommon for patients to have symptoms for months prior to clinicians making the diagnosis with the median duration from symptom onset to diagnosis being about 4 months [2].

Classical presentation of JDM includes the pathognomonic dermatologic findings of a heliotrope rash and/or Gottron papules. These findings along with symmetric proximal muscle weakness should raise suspicion of JDM. Previously the only available diagnostic criteria were based on criteria published in 1975 by Bohan and Peter who discussed the following five diagnostic criteria: classical skin rash, elevated muscle enzymes, proximal muscle weakness, as well as characteristic changes seen on electromyogram (EMG) and muscle biopsy [3, 4]. While these criteria defined the basis for diagnosis of JDM, these criteria require invasive testing not always readily available, and with the advancement of antibody testing, we have begun recognizing variable presentations of JDM that may not meet these diagnostic criteria. This was addressed in 2017 when the European League against Rheumatism and American College of Rheumatology established classification criteria for IIM’s and the major subgroups. These criteria attempt to score patients based on whether or not muscle biopsy results are available and account for age of symptom onset, muscle weakness, skin manifestations, dysphagia or esophageal dysmotility, elevated muscle enzymes, and presence of Anti-Jo-1 antibody [5]. These new data-driven, highly specific, and sensitive criteria for IIM and the major subgroups allow clinicians to confidently diagnose myopathies, including JDM, which should allow for earlier diagnosis and management. Earlier treatment is paramount in avoiding potential life-threatening complications of the IIM.

Macrophage activation syndrome (MAS) is an acute life-threatening systemic inflammatory process with some case reviews estimating around 20% mortality [6]. While recognized as a life-threatening inflammatory process, the diagnostic criteria for MAS have been difficult to clearly define. There are common clinical features including fever, hepatosplenomegaly, lymphadenopathy, and coagulopathy along with some classic lab markers including elevated liver enzymes, hyperferritinemia, and cytopenias. Many of the clinical and lab criteria were drawn from hemophagocytic lymphohistiocytosis (HLH) which can be broken down into primary and secondary HLH. Macrophage activation syndrome is considered a form of secondary HLH, specifically triggered by autoimmune or rheumatologic diseases. While closely related, the diagnostic criteria for HLH were developed primarily for the genetic or primary form of HLH. Only recently have diagnostic criteria for MAS specifically been developed in the setting of systemic juvenile idiopathic arthritis (sJIA) [7]. In recent years MAS has become more recognized as a complication secondary to other inflammatory diseases. Recent literature has shown that MAS may not be as rare a complication of JDM as previously believed and has been described in multiple case reports [8, 9].

Here we describe a patient with a clinically difficult to diagnose rare subset of anti-MDA5 JDM who progressed rapidly to treatment-refractory MAS. During her hospital course, our patient had significant multi-organ system involvement including atrial fibrillation without rapid ventricular response. To our knowledge, only one other case report has been described in the literature of this occurring in the pediatric population [10].

## Case presentation

Our patient is a 14-year-old female with a history of a non-functioning micropituitary adenoma found incidentally during imaging for concussion, chronic constipation, reflux, anxiety, and depression presenting with complaints of persistent daily fevers for 6 weeks and a sixteen-pound weight loss over 2 months. A complete review of her medical history was significant for a family history of an aunt with type 1 diabetes, and a cousin with Sjogren’s syndrome. Two months prior to presentation she was diagnosed with Hand, Foot, and Mouth (HFM) disease. After brief resolution of HFM symptoms, she then began having daily fevers with the highest temperature of 104 °F. Review of systems was significant for daily fevers, cough, difficulty breathing, chest pain, headaches, sore throat, polyarthralgia, myalgias, nausea, and loss of appetite leading multiple providers to evaluate her for Covid-19. Previous workup had been significant for erythrocyte sedimentation rate (ESR) of 48 mm/hr., hemoglobin of 9.1 g/dL, absolute lymphocyte count of 600/mcL, lactate dehydrogenase (LDH) 696, AST 281 U/L, and ALT 157 U/L. Blood and urine cultures were negative. She had multiple negative Covid-19 PCRs no evidence of a source of infection. Chart review on admission was significant for a sixteen-pound unintentional weight loss over 2 months, dropping from the 54th to 21st percentile on her weight growth curve with preservation of her height over that time frame. Her exam was remarkable for mild weakness, no overt arthritis, and dry plaques over her MCPs and elbows.

Initial broad differential for prolonged daily fevers of unknown origin and weight loss included but was not limited to infectious, malignant, or autoimmune etiologies. During the extensive workup, she continued to have multi-system involvement making diagnosis of the underlying etiology difficult. Her extensive infectious disease workup showed no evidence of an underlying infectious etiology including a negative next-generation sequencing of microbial cell-free DNA evaluation. Workup for malignancy was negative including bone marrow evaluation (Table 1).

**Table 1:** Extensive lab evaluation for infection, malignant, and rheumatologic diseases as underlying etiology of MAS

**Initial Lab Evaluation**	
**Labs**	**Result (Classification)**
Blood/Urine/Fungal/Anaerobic/Stool Cultures	Negative
Covid-19 PCR and IgG & IgM	Negative
EBV/CMV IgG & IgM	Negative
Hepatitis A/B/C- Ab Panel	Nonreactive
Leptospira Culture & Ab	Negative
*Bartonella Henselae*/Quintana- Ab IgG & IgM	Negative
*Rickettsia Typhi*- Ab IgG & IgM	Negative
Legionella- Ag(urine)	Negative
Mycoplasma pneumonia- Ab IgM	Negative
HIV-1 + 2 Ab/HIV P24 Ag	Negative
Microbial/viral/fungal/protozoal free DNA	No statistically significant levels detected
Coccidioides-Abs IgG &IgM	< 0.2 (Normal)
Blastomyces- Ab	Negative
Histoplasma Capsulatum-Ag(urine)	Negative
Cryptosporidum/Giardia- Ag(stool)	Negative
Enterovirus RNA (stool)	Negative
**Blood Smear**	**Anemia, thrombocytopenia, neutropenia, lymphopenia**
Bone Marrow Biopsy (smear)	No evidence of malignancy or HLH
Bone Marrow Biopsy (FISH)	All test values within normal limits
Bone Marrow Biopsy (Leukemia/Lymphoma panel)	No evidence of B/T cell lymphoproliferative disorder
Bone Marrow Biopsy (Cytogenetics)	No clonal abnormalities
ANA	Negative
RF	< 15 (Normal)
Cyclic Citrullinated Peptide IgG & IgA	9 (Normal)
Centromere B-Ab	< 0.2 (Normal)
SCL-70 Extractable Nuclear-Ab	< 0.2 (Normal)
Ribonucleoprotein Extractable Nuclear-Ab	0.2 (Normal)
Smith Extractable Nuclear-Ab	< 0.2 (Normal)
**SS-A-Ab**	**1.5 (High)**
SS-B-Ab	< 0.2 (Normal)
DNA DS-Ab	< 1 (Normal)
**Creatinine Kinase**	**217 (High)**
**Aldolase**	**14.9 (High)**
**Smooth Muscle-Ab**	**87 (High)**
Cardiolipin/Beta-2 Glycoprotein IgG & IgM	Negative
Liver-Kidney-Ab	0.8 (Normal)
Mitochondrial- Ab	< 20.0 (Normal)
Parietal Cell- Ab	1.2 (Normal)
**Von Willebrand Factor-Ag**	**239 (High)**

**Fig. 1:**
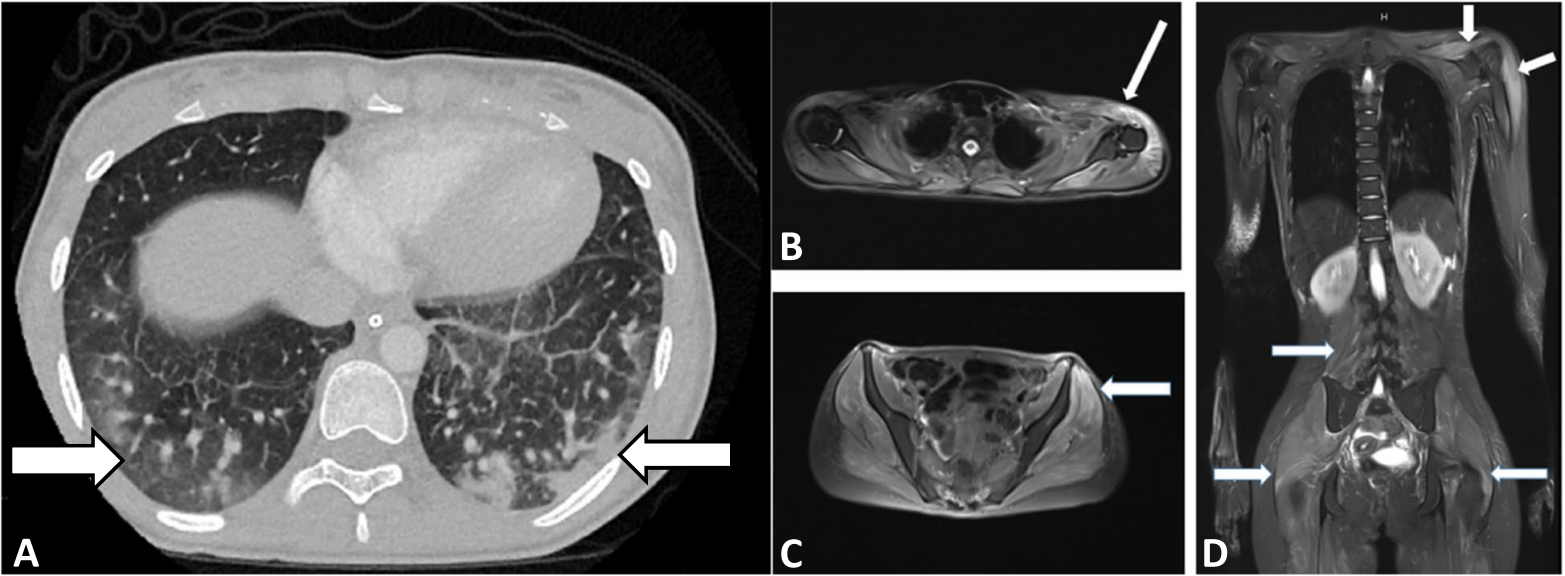
**A** CT chest showing bilateral posterior lower lobe consolidations worse on the left **B.** T2 fat saturated MRI showing myositis of the left deltoid **C**. Myositis of the gluteus medius and minimus **D.** Myositis of the shoulders

The evaluation confirmed suspicion for autoimmune disease with creatine kinase (CK) of 217 U/L, aldolase 14.9 U/L, ferritin 1641 ng/mL, anti-SS-A/Ro antibody 1.5 AI, anti-smooth muscle antibody 87 Units, and von Willebrand factor antigen 239%, however without any confirmatory results for a specific disease process. Throughout her workup, she continued to have persistent complaints of GI discomfort and reflux treated with a PPI. She had concerning pulmonary involvement and complaints of difficulty breathing. Initial imaging showed significant consolidations, ground-glass opacities, and interstitial involvement (Fig. 1 a).

On diagnostic bronchial alveolar lavage there was concern for mucosal friability and bleeding in small airways. Her PFTs showed moderate ventilatory deficits in a mixed restrictive/obstructive pattern. Follow-up imaging and PFTs showed persistent interstitial lung disease and mixed ventilatory deficit but the patient did not require supplemental oxygen. These findings of interstitial lung disease in conjunction with her family history of Sjogren’s and positive anti-SSA/Ro raised our clinical concern for primary Sjogren’s syndrome versus an IIM given the elevated muscle enzymes.

Due to significant pulmonary findings, lab abnormalities with worsening transaminitis, and decreased likelihood of infection the decision was made to begin initial treatment with steroids. On hospital day 11 patient was started on a three-day pulse of 1000 mg IV methylprednisolone, followed by Prednisone 50 mg daily orally (1.2 mg/kg/day). The patient showed significant clinical improvement as well as laboratory improvement of her transaminitis and pancytopenia. She developed significant thrush, which was resistant to oral nystatin and required systemic treatment with caspofungin. Unfortunately, she had recurrence of her fevers on hospital day 15 as well as declining neuro-psychological features with acutely worsening anxiety and developmental regression, prompting repeat pulse doses of methylprednisolone IV 500 mg, followed by 2 mg/kg/day divided twice daily. She also had cardiac abnormalities which persisted despite steroid administration. On initial presentation, given complaint of chest pain, an EKG was obtained showing first-degree heart block with global low voltages but her echo was without any abnormalities. On hospital day 18 she had new complaints of intermittent chest pain at rest with a repeat EKG showing prolonged QTc and T wave inversions in left lateral leads (I, V5, V6). Repeat echo again did not show any abnormalities, including no evidence of coronary artery dilation, myocarditis, or pericarditis. The patient was placed on telemetry given abnormal changes in EKGs.

Due to persistent symptoms, repeat PFT’s showing continued interstitial lung disease, and worsening of lab values, the patient was started on mycophenolate mofetil (MMF) at 600 mg/m2 twice daily on day 20 with high dose IV steroids. MMF was chosen as a means of targeting the rheumatic disease-related interstitial lung disease and its relatively lower side effect profile when compared to other immunosuppressants. Subsequently, a T2 fat saturated MRI of the chest and pelvis was obtained and showed myositis of shoulders, paraspinal muscles, and proximal thighs (Fig. 1 b-d) increasing concern for myositis and prompting a muscle biopsy.

**Fig. 2:**
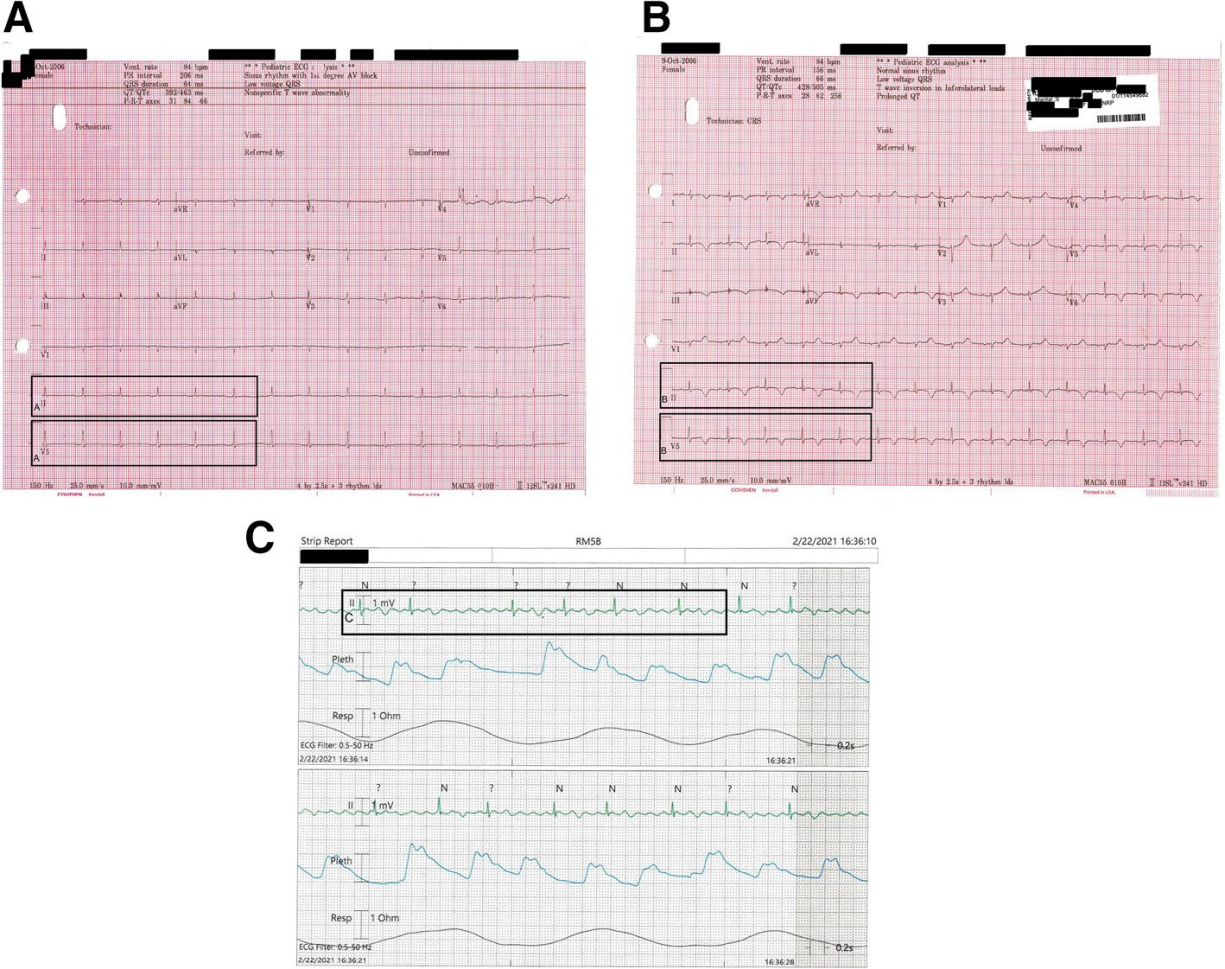
**A** EKG at baseline with low voltage and 1st-degree heart block **B.** EKG with new T-wave inversion in left lateral leads **C.** EKG with Progression atrial flutter/fibrillation with slow ventricular response

Despite MMF and high dose steroids, the patient’s labs continued to worsen and on day 21 was noted to be in an irregularly irregular rhythm without discernable P waves and a heart rate of 60–70 diagnosed as atrial fibrillation without rapid ventricular response (Fig. 2).

Her echo showed possible coronary dilation so a cardiac MRI was performed, showing no abnormalities. Her rhythm normalized without intervention. Her maximum troponins were 0.030 ng/mL. Due to continued symptoms, clinical progression now with cardiac involvement, and again worsening labs the decision was made to begin IVIG 70 g and anakinra 2 mg/kg twice daily SQ for treatment of MAS complicating a likely IIM on day 25.

**Fig. 3:**
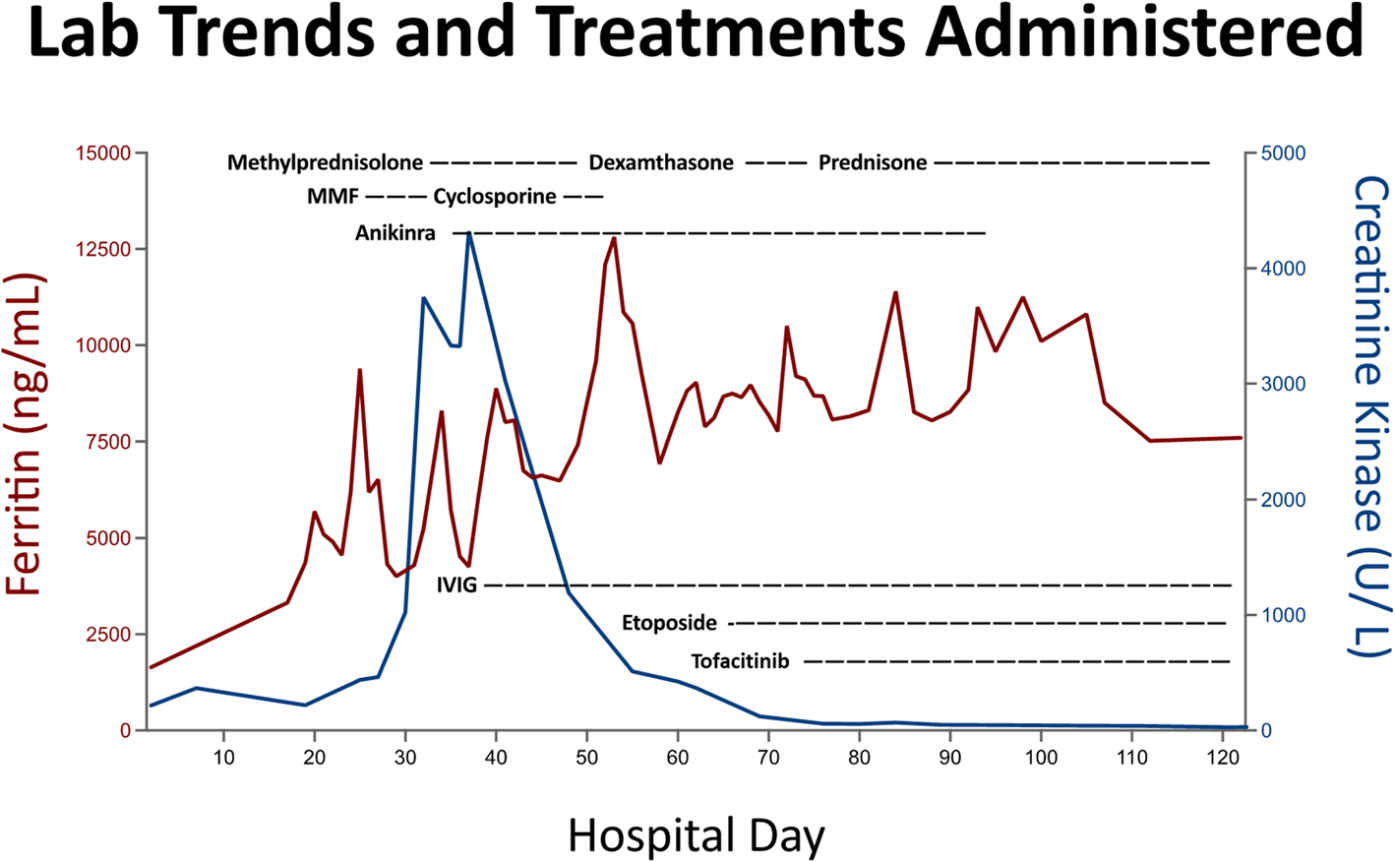
Lab trends and immunosuppressant treatments administered during the hospital course. Ferritin was used as a marker of MAS response and evidence of systemic inflammatory response while creatinine kinase (CK) was used to monitor response of myositis

Shortly after the initiation of anakinra the confirmatory myositis antibody panel sent at the time of hospital presentation returned positive for anti-MDA5 and anti-SSA-52 antibodies, which are seen in a distinct phenotype of juvenile IIM. Despite the use of anakinra her underlying inflammation progressed. At this time the patient was transferred to a medical facility with pediatric rheumatology capabilities. Her MMF was discontinued, Anakinra was increased in intervals up to a maximum of 160 mg every 6 h IV (15 mg/kg/day), and IV cyclosporine 3–5 mg/kg/day divided every 12 h was started with the dose adjusted based on the trough level, along with continued IVIG and IV methylprednisolone. As her ferritin continued to climb to levels greater than 10,000 ng/mL and she developed posterior reversible encephalopathy syndrome, her cyclosporine was discontinued. She was transitioned to etoposide 150 mg/m2 twice weekly for 2 weeks, then weekly for 6 additional weeks and dexamethasone 10 mg/m2 divided twice daily in addition to continued Anakinra and IVIG (Fig. 3). Tofacitinib was added at 5 mg orally twice daily. This combination (Etoposide, Dexamethasone, Anakinra, IVIG, and Tofacitinib) was successful as indicated by significant clinical improvement in her multi-system organ involvement.

Anakinra was weaned and her IV steroid was transitioned to oral prednisone while continuing tofacitinib. At the time of this report she is stable on low dose prednisone of 5 mg daily, tofacitinib 10 mg twice daily, and IVIG.

## Discussion and conclusions

**Fig. 4:**
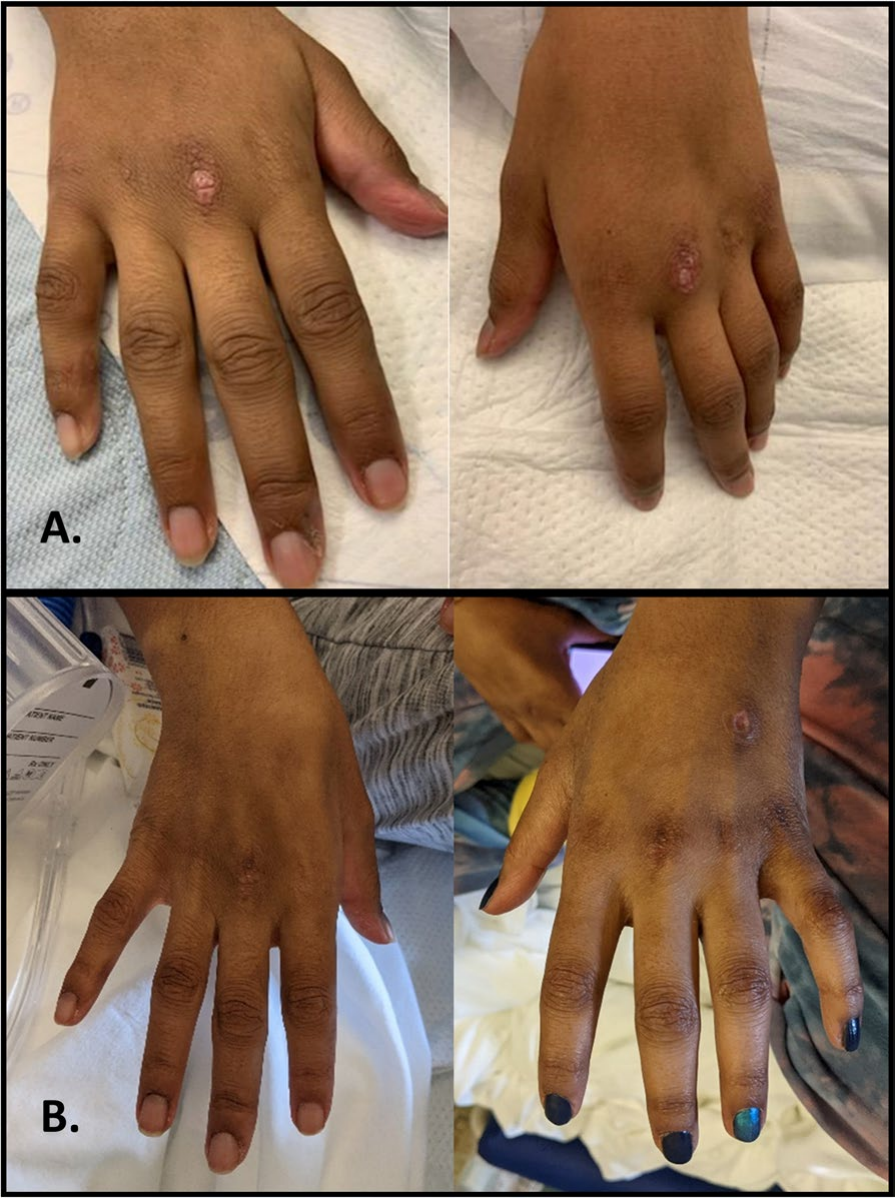
**A** Dorsum of patient’s hands at time of presentation with two small ulcerative lesions on each hand. **B** Dorsum of patient’s hands approximately 3 months after initiation of immunosuppressive treatments for JDM and MAS

Our patient demonstrates the complexity in evaluating rheumatologic conditions with multi-organ-system involvement, ultimately diagnosed with MDA5 antibody positive JDM complicated by MAS. Complexities of this case include limited skin findings isolated only to two ulcerative lesions on the dorsum of her hands, initially thought to be the healing lesions of her prior HFM disease but in retrospect were likely the initial presenting rash of her underlying JDM (Fig. 4).

Her multisystem involvement in the setting of negative infectious and malignancy evaluations was supportive of an autoimmune disease. Her elevated transaminases, LDH, CK, and aldolase were consistent with myositis and potentially an IIM, however, Sjogren’s syndrome can also present with myositis and was considered initially given her significant cardiopulmonary involvement and positive anti-SSA antibody. To further elucidate the underlying rheumatologic etiology, MRI was utilized to demonstrate proximal muscle inflammation. Myositis antibodies and a muscle biopsy were also obtained for their diagnostic and prognostic utility, ultimately confirming the diagnosis of anti-MDA5 positive JDM.

MAS is an uncommon life-threatening complication secondary to an underlying inflammatory disease state. In pediatrics, the most commonly associated underlying rheumatologic disease is systemic juvenile idiopathic arthritis (sJIA) but has been reported with increased frequency in multiple other rheumatologic conditions including systemic lupus erythematosus, Kawasaki disease, and JDM [11, 12], all of which were evaluated for in our patient. The underlying rheumatologic process leads to excessive activation and expansion of macrophages and T-cells leading to a further overwhelming inflammation that can involve every organ system. It can be difficult to make the diagnosis of MAS as it often resembles the multisystem organ dysfunction that can be seen with the respective underlying disease processes. In addition, there has been a lack of consensus on strict clinical or laboratory features to define the diagnosis. In 2016, EULAR/ACR approved criteria for making the diagnosis of MAS in pediatrics patients with sJIA [7], however, this has not yet been validated across other underlying systemic diseases. HLH is a similar disease process, defined by uncontrolled inflammation secondary to underlying genetic mutations in perforin, infection, or malignancy. HLH diagnostic criteria include fever, splenomegaly, and hyperferritinemia [13]. The formal HLH criteria are often insensitive for MAS and can lead to under diagnosis of MAS [14]. Our patient did have an evaluation for HLH early on in her hospitalization, meeting very few of the criteria for diagnosis.

While awareness and recognition of MAS as a clinical syndrome has increased, the majority of the literature describes it in association with more common underlying diseases, specifically sJIA in children. Based upon an international consensus survey, the most common clinical and laboratory features of MAS secondary to sJIA in order of frequency are decreasing platelet counts, hyperferritinemia, increasing liver enzymes, falling leukocyte count, persistent continuous fever ≥38 °C, falling erythrocyte sedimentation, hypofibrinogenemia, and hypertriglyceridemia [7]. Previous reviews of the literature have discussed some variation in clinical presentation of MAS dependent on the underlying rheumatologic condition [12]. A previous report of a 4-year-old with JDM progressing to MAS described that while there may be some similarities, the cytokine profile of MAS secondary to JDM differs from that of MAS secondary to sJIA [10], which may explain the variable clinical manifestations. The potential for rheumatologic conditions to have multi-organ involvement and progression to MAS with unclear clinical criteria for diagnosis and with variable clinical presentations leads to difficulty and delay in diagnosis. While it is known that MAS is a possible complication of JDM it is still unclear why this occurs in certain cases. It is possible that the delay in diagnosis and adequate control of the underlying JDM led to the hyper-inflammatory state, however, it is also possible that our patient’s uncommon subtype of anti-MDA5 positive JDM may have left her more predisposed to progressing to MAS.

A unique aspect of our case was the cardiac arrhythmias that our patient experienced. The cardiac manifestations in MAS are often described as myocarditis or pericarditis secondary to the underlying inflammation, albeit very few of these descriptions exist in the literature [13, 15]. While cardiac complications of JDM are well known, this is often systolic and diastolic dysfunction with increased risk of ischemic heart disease later in life, in addition to reports of pericarditis and myocarditis [16]. Our patient had electrical conduction abnormalities. While there are descriptions of rare conduction abnormalities in children newly diagnosed with JDM, our patient’s atrial fibrillation without rapid ventricular conduction has not been described [17]. It has been postulated that the conduction abnormalities found in JDM may have to do with autonomic dysregulation [18]. Her cardiac conduction abnormalities likely represented her progressive disease state. We recommend that others have low thresholds to obtain EKG evaluations in patients with progressive JDM and MAS.

## Conclusion

JDM in the pediatric patient is rare, as is MAS. In patients with complex rheumatologic conditions and lack of response to treatment, it is important to continually assess the patient’s clinical status with MAS in mind, as this may change the treatment approach. Without proper recognition of this progression, patients can have a significant delay in diagnosis leading to life-threatening consequences.

## Data Availability

Patient data used during this report are available from corresponding author on reasonable request.

## Abbreviations

MAS: Macrophage Activation Syndrome
JDM: Juvenile Dermatomyositis
IIM: Idiopathic Inflammatory Myopathies
MMF: Mycophenolate Mofetil
sJIA: systemic Juvenile Idiopathic Arthritis
HLH: Hemophagocytic Lymphohistiocytosis
IV: Intravenous
IVIG: Intravenous immune globulin
